# Aquatic plant surface as a niche for methanotrophs

**DOI:** 10.3389/fmicb.2014.00030

**Published:** 2014-02-03

**Authors:** Naoko Yoshida, Hiroyuki Iguchi, Hiroya Yurimoto, Akio Murakami, Yasuyoshi Sakai

**Affiliations:** ^1^Division of Applied Life Sciences, Graduate School of Agriculture, Kyoto UniversityKyoto, Japan; ^2^Center for Fostering Young and Innovative Researchers, Nagoya Institute of Technology, NagoyaAichi, Japan; ^3^Kobe University Research Center for Inland SeasAwaji, Hyogo, Japan; ^4^Japan Science and Technology Agency, CRESTAwaji, Hyogo, Japan; ^5^Advanced Low Carbon Technology Research and Development Program, Japan Science and Technology AgencyTokyo, Japan

**Keywords:** methanotroph, aquatic plants, *pmoA*, methane monooxygenase, methane sink

## Abstract

This study investigated the potential local CH_4_ sink in various plant parts as a boundary environment of CH_4_ emission and consumption. By comparing CH_4_ consumption activities in cultures inoculated with parts from 39 plant species, we observed significantly higher consumption of CH_4_ associated with aquatic plants than other emergent plant parts such as woody plant leaves, macrophytic marine algae, and sea grass. *In situ* activity of CH_4_ consumption by methanotrophs associated with different species of aquatic plants was in the range of 3.7–37 μmol·h^−1^·g^−1^ dry weight, which was ca 5.7–370-fold higher than epiphytic CH_4_ consumption in submerged parts of emergent plants. The qPCR-estimated copy numbers of the particulate methane monooxygenase-encoding gene *pmoA* were variable among the aquatic plants and ranged in the order of 10^5^–10^7^ copies·g^−1^ dry weight, which correlated with the observed CH_4_ consumption activities. Phylogenetic identification of methanotrophs on aquatic plants based on the *pmoA* sequence analysis revealed a predominance of diverse gammaproteobacterial type-I methanotrophs, including a phylotype of a possible plant-associated methanotroph with the closest identity (86–89%) to *Methylocaldum gracile*.

## Introduction

Methane (CH_4_) is the second most important greenhouse gas after CO_2_, and it accounts for 20–30% of the contribution of greenhouse gases to global warming (Solomon et al., 2007). Its concentration has rapidly increased by 2.5-fold since 1750 from approximately 700 to 1775 ppbv in 2005 (Solomon et al., 2007), although annual increases have varied. For long-term forecasting and control of atmospheric CH_4_, it is necessary to understand the CH_4_-flux, or the balance of sources and sinks in the environment. One of the most important processes of CH_4_ production is methanogenesis by Archaea. On the other hand, the largest CH_4_ sink is the dispersion (>80%) via reaction with hydroxyl-radicals in the troposphere, and the second sink is diffusion into the stratosphere. Consumption of CH_4_ by methanotrophs is the main biological CH_4_ sink, and is considered to contribute significantly to CH_4_ mitigation under both aerobic and anaerobic conditions (Conrad, 2009; Borrel et al., 2011). Active CH_4_ consumption is considered to occur at the oxic-anoxic interface. However, CH_4_ consumption in such environments is overlooked when the emission of CH_4_ is balanced against consumption. Local CH_4_ sinks have recently been investigated in various environments such as surface layers of wetlands, paddy fields, and sediments (Conrad, 2009). For example, more than 90% of the potentially emitted CH_4_ was consumed in the surface layer of lake sediment (Frenzel et al., 1990). Some important ecological CH_4_ sinks might be unaccounted for or not discovered.

Microbial consumption of CH_4_ is mainly conducted by methanotrophic bacteria. Methanotrophic bacteria belong to the phyla *Proteobacteria* (Hanson and Hanson, 1996), *Verrucomicrobia* including extremely acidophilic methanotrophs isolated from volcanic habitats (Op den Camp et al., 2009), and the candidate division NC10. NC10 includes a candidate oxygenic methanotroph that oxidizes CH_4_ by using O_2_ produced from nitrite (Ettwig et al., 2010). Among those methanotrophs, proteobacterial methanotrophs have been frequently detected as active CH_4_ utilizers in terrestrial environments, and grouped into the Gammaproteobacteria and Alphaproteobacteria. Gammaproteobacterial type I methanotrophs develop intracytoplasmic membranes (ICMs) as bundles of vesicular discs for CH_4_ oxidation and use the ribulose monophosphate pathway for formaldehyde fixation, while type II methanotrophs, which are members of the Alphaproteobacteria, possess ICMs composed of paired peripheral layers and utilize the serine pathway for formaldehyde fixation. The traditional type I and type II classification of methanotrophs were postulated based on these physiological characteristics. However, recent isolation of new methanotrophs revealed many exceptions to the traditional classification, giving more updated view on methanotrophs ecology, physiology, and phylogeny (Semrau et al., 2010; Borrel et al., 2011; Stein et al., 2012). Methane oxidization is catalyzed by two types of methane monooxygenases (MMO), soluble cytoplasmic MMO (sMMO), and membrane-bound particulate MMO (pMMO). While sMMO is found in a subset of methanotrophs, pMMO is present in all methanotrophs except *Methylocella* and *Methyloferula* (Dedysh et al., 2000; Dedysh, 2009; Vorobev et al., 2011). Therefore, the gene *pmoA*, encoding the beta subunit of pMMO, is often used as a biomarker to specifically detect aerobic methanotrophs. The sequences of *pmoA* are evolutionally conserved and reflect the 16S rRNA gene-based phylogeny of methanotrophs (McDonald et al., 2008).

Plant surfaces, not only in the rhizosphere, but also the emergent parts of plants, or phyllosphere, have recently attracted attention as sites for CH_4_ emission. Some plants have been reported to produce CH_4_ coupled with photosynthesis and emit CH_4_ from plant leaves (Keppler et al., 2006); such CH_4_ emissions account for 6% of the total CH_4_ emission on earth (Conrad, 2009). As is commonly observed in environments near CH_4_ emission-sources, a local CH_4_ sink in plants has also been reported for mosses (Raghoebarsing et al., 2005), rice roots, and emergent and aquatic plants (Heilman and Carlton, 2001; Sorrell et al., 2002).

CH_4_ is also expected to be consumed by plant-associated methanotrophs, which we have recently enriched and identified from various plant parts together with methanol-utilizing bacteria (Iguchi et al., 2012). The latter are well known as common inhabitants of the phyllosphere (Lidstrom, 2006; Knief et al., 2011; Vorholt, 2012). These findings suggested a possible contribution of methanotrophs to oxidation of CH_4_ in the phyllosphere. Therefore, studies on the interaction between plants and methanotrophs will provide useful information to understand and control the carbon cycle. However, such methanotroph-plant interactions have been investigated in very few plants, such as rice (Shrestha et al., 2008; Pfluger et al., 2011; Stein et al., 2012) and *Sphagnum* mosses (Raghoebarsing et al., 2005; Kip et al., 2011).

In this study, the local CH_4_ sink in plants was evaluated using plant parts sampled from various environments to determine the potential of methanotrophs to consume CH_4_ in different species of plants. After comparing the CH_4_-consuming activities of 39 different plant species, we discovered high CH_4_ consumption by methanotrophs on aquatic plants. Furthermore, methanotrophs in aquatic plants were quantified and identified based on *pmoA* sequence analysis.

## Materials and methods

### CH_4_ consumption in cultures inoculated with plant parts

As a first step to determine what kinds of plants have associations with CH_4_ consuming methanotrophs, CH_4_ consumption by plant samples was assayed by analyzing the changes of CH_4_ concentration in cultures inoculated with various plant parts listed in Supplemental Table 1. About one gram of plant material was placed into a glass-vial (60 mL capacity), which was filled with 20 ml of autoclaved mineral NMS medium (Whittenbury et al., 1970) supplemented with trace vitamins and trace elements (Yoshida et al., 2007). Submerged parts of emergent aquatic plants were used for incubations after washing by shaking in distilled water three times to remove soil. The incubation vials were closed with Teflon-coated butyl rubber caps. CH_4_ (5% v/v) was added to the vial by syringe and vials were incubated for 14 days at 28°C with shaking.

### CH_4_ consuming activities of methanotrophs in aquatic plants

**Figure 1:**
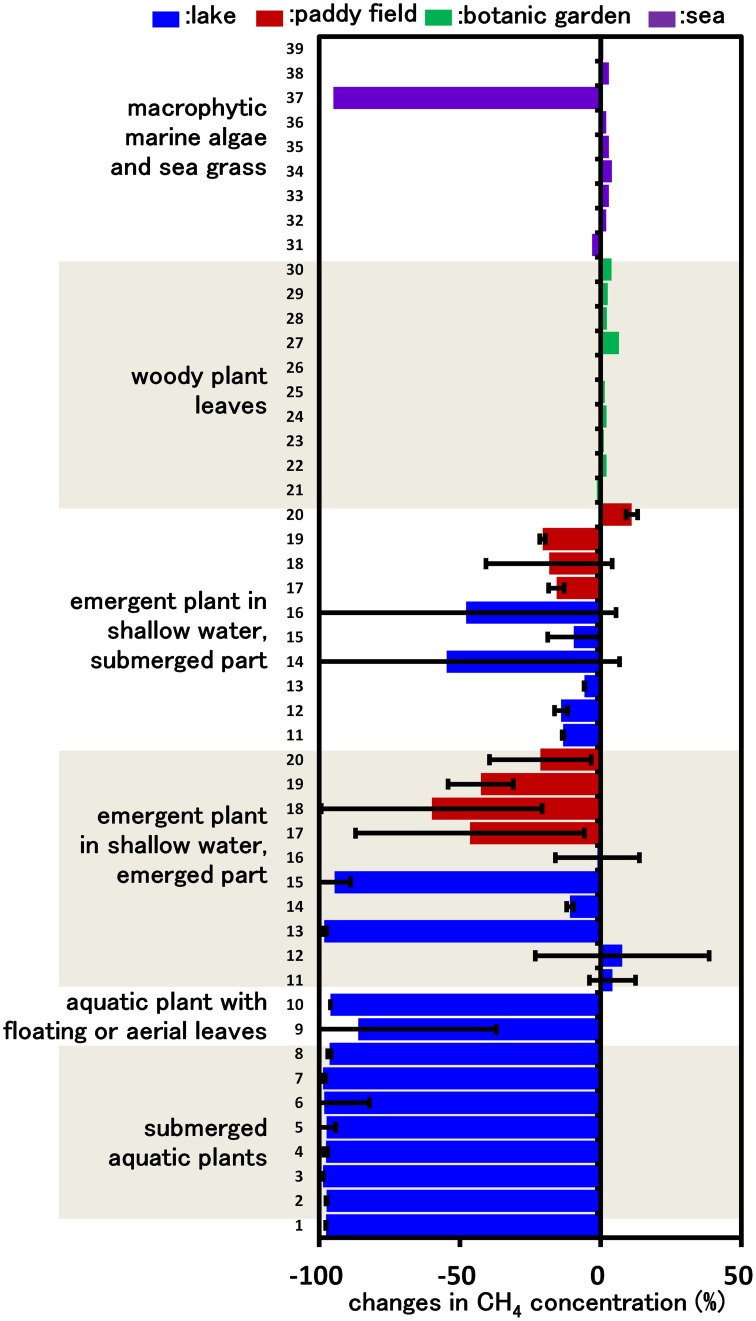
**CH_4_ consumption in cultures inoculated with various plant parts.** Columns indicate the changes in CH_4_ concentration after incubation for 14 days in vials containing mineral medium amended with 5% CH_4_ and plant parts. Each column indicates a single determination inoculated with each plant species (listed in Supplemental Table 1). Data for emergent and submerged aquatic plants represent the mean ± SD from three different glass bottles independently prepared in parallel, while data for tree leaves and sea weed are single determinations.

Eight submerged aquatic plants and two aquatic plants with floating or aerial leaves (Figure 1) were sampled from a shallow eutrophic sub-basin (614 km^2^ of surface area and 3.5 m average depth) of Lake Biwa in Japan (35°4′ 32″N, 135°56′ 5″E), where the area covered with aquatic plants amounted to 52% of the total surface area in the basin in 2000 (Hamabata and Kobayashi, 2002). To analyze CH_4_ consumption activities by the aquatic plants, 5 g of plant material (wet weight) from each macrophyte was placed into 120 mL glass vials. Rice, *Oryza sativa* spp. *japonica*, cultivator Koshihikari, which had been growing for about 2 months and was 30 cm in height, was analyzed for comparison. The glass vials were supplemented with 1% (v/v) CH_4_ and closed with butyl-lubber caps. The vials were incubated for 30 h at 25°C in the dark and the CH_4_ in the headspace was analyzed every hour by using GC-FID as reported previously (Iguchi et al., 2011). Ten mL of lake water was used instead of aquatic plants to compare the CH_4_ consumption activities with those of aquatic plants. Aquatic plants and rice were washed by shaking three times in lake water and flood water from the rice field, respectively.

### DNA extraction from water plant material

For analysis of methanotrophic microbial communities in the aquatic plants, approximately 0.5 g of plant material (wet weight) from each plant were used for DNA extraction. DNA was extracted from the aquatic plants by using an ISOIL DNA extraction kit (NIPPON GENE, Tokyo, Japan) according to manufacturer's instructions.

### qPCR targeting *pmo*A

qPCR targeting the *pmoA* gene using primers A189 and A682 (Holmes et al., 1995) was performed with a LightCycler FastStart DNA Master SYBR green I kit (Roche Molecular Biochemicals, Indianapolis, IN) and the LightCycler system (Roche Applied Science, Indianapolis, IN) as described previously (Yoshida et al., 2009). The PCR profile consisted of preheating at 95°C for 10 min and 40 cycles of denaturation at 95°C for 15 s, annealing at 60°C for 10 s, and extension at 72°C for 20 s. The fluorescence signal was detected at 72°C during each cycle, and a melting curve was obtained by heating the product to 95°C and cooling it to 40°C. The calibration curves were graphed and the copy numbers of *pmoA* were calculated with LightCycler software version 3.5 (Roche Diagnostics) using serial dilutions of the *pmoA* amplicons from *Methylococcus capsulatus* Bath as a standard. The PCR efficiency was 90%, which was calculated from qPCR using serially diluted standard samples prepared from the *pmoA* amplicons of *Methylococcus capsulatus* Bath. Melting curve analyses demonstrated the absence of primer dimer formation and non-specific amplifications in all samples facilitating the use of 72°C as temperature of recording fluorescence.

### Clone library of *pmo*A amplicons from aquatic plants

The extracted DNA samples were used as templates for amplification of *pmoA* by using two *pmoA*-specific primer sets, A189 and A682 (Holmes et al., 1995) and A189 and mb661 (Costello and Lidstrom, 1999). PCR using KOD Fx Neo (TOYOBO, Osaka, Japan) was performed by preheating the mixture to 94°C for 2 min, followed by 30 cycles of denaturation at 98°C for 10 s, annealing at 55°C for 30 s, and extension at 74°C for 30 s. The *pmoA* amplicons from aquatic plants were cloned by using a pCR^®^8/GW/TOPO^®^ TA cloning kit (Invitrogen, Carlsbad, CA, USA) and sequenced using a BigDye Terminator v3.1 cycle sequencing kit and an ABI 3130 Genetic Analyzer (Applied Biosystems). The clones were chimera-checked by using chimera detection software, Bellerophon (Huber et al., 2004) and classified to operational taxonomic units (OTUs) having <0.07 of the evolutional distance (e.d.) in amino acid sequences by using Mothur (Schloss et al., 2009). The phylogenetic tree of the OTUs and relatives was drawn by the neighborhood joining method using MEGA5.1 (Tamura et al., 2011).

### Enrichment and isolation of methanotrophs from aquatic plants

A piece (about 1 cm^2^) of aquatic plant material was inoculated into a glass-vial (30 mL capacity) containing 5 ml of NMS or AMS medium (Whittenbury et al., 1970) and 10% CH_4_ (v/v) as described above. The cultures were incubated at 28°C with shaking and subcultured in fresh medium when CH_4_ in the headspace was consumed. After 3–4 serial transfers, the cultures were serially diluted and spread onto 1.5% agar plates of NMS or AMS medium. The agar plates were incubated for 2–4 weeks at 28°C in a jar filled with a CH_4_/air mixture. About 100 colonies were picked and subcultured from each culture. The colonies were purified by repeating agar cultivation until a single colony morphotype was obtained. The isolates were phylogenetically identified by sequencing 16S rRNA and *pmoA* genes, which were amplified from cell lysates of the isolates (Yoshida et al., 2007).

### Nucleotide sequence accession numbers

The nucleotide sequence data reported in this paper have been deposited under DDBJ/EMBL/GenBank (http://www.ddbj.nig.ac.jp/Welcome-j.html) accession no. AB844797–AB845152, AB845287–AB845309, and AB845153–AB845175.

## Results

### CH_4_ consumption in cultures inoculated with various parts of plants

Our previous study indicated that methanotrophs inhabit emergent parts of various plant surfaces (Iguchi et al., 2012). In this work, we aimed to determine what types of plant could potentially serve as a niche for methanotrophs. Based on the assumption that CH_4_ consumption is only carried out by methanotrophic bacteria, we measured CH_4_ consumption in cultures inoculated with plant parts taken from various environments, thereby evaluating CH_4_ consumption activity by plant-associated methanotrophic consortia. After 14 days of incubation, none of the cultures inoculated with woody plant leaves showed detectable consumption of CH_4_. In contrast, cultures inoculated with several emergent plants, macrophytic marine algae and all aquatic plants consumed significant amounts of CH_4_ (Figure 1). In the emergent grass cultures, the cultures of emergent parts consumed higher amounts of CH_4_ than those of submerged parts. As a result of this screening, we found that aquatic plants showed a constant high CH_4_-consuming activity, and therefore the aquatic plants were subjected to further investigation.

### CH_4_ consumption by aquatic plants

**Figure 2:**
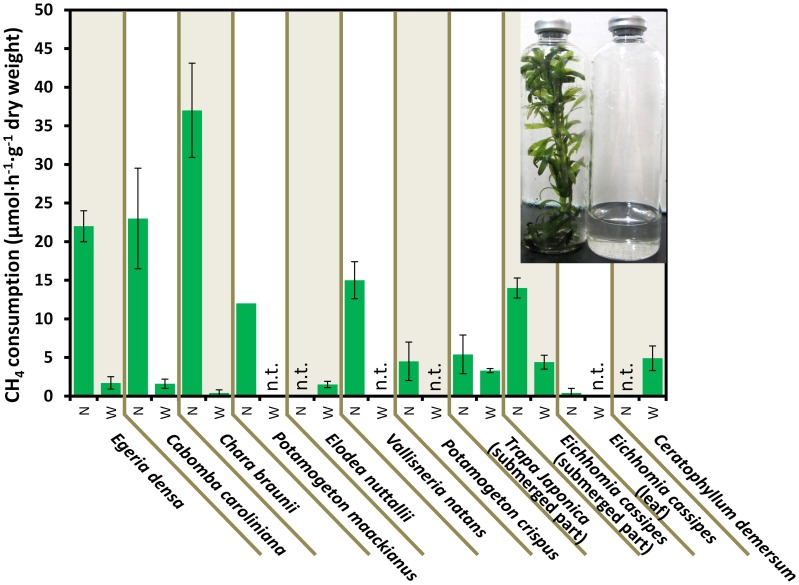
**CH_4_ consumption in the aquatic plants.** Data represent the mean ± SD from three different glass bottles independently prepared in parallel, except for the single determination with *P. maackianus*. The photo shows the incubated *E. densa* (left) and lake water (right) in bottles. N and W indicate unwashed native samples and samples washed by shaking three times in lake water, respectively. “n.t.” is “not determined.”

Figure 2 shows the CH_4_ consumption by eight submerged aquatic plants and two aquatic plants with floating or aerial leaves. Within 30 h of incubation at 25°C, all 10 aquatic plants consumed significant amounts of CH_4_, in contrast to the vial containing lake water without aquatic plants, which did not consume CH_4_ within 30 h (data not shown). The CH_4_ consumption by aquatic plants was variable among the aquatic plants and sample preparations, exhibiting 3.7–37 μmol·h^−1^·g^−1^ dry weight of CH_4_ consumption in native submerged samples. In the control using rice as an emergent plant, the CH_4_ consumption was lower than that of aquatic plants with values of 0.018 ± 0.08, 0.29 ± 0.22, and 0.37 ± 0.03 μmol·h^−1^·g^−1^ dry weight for leaves, stems and washed roots, respectively. These results showed that aquatic plants had much higher CH_4_ consumption activity than the emergent plants.

CH_4_ consumption by aquatic plants declined to 0.45–4.4 μmol·h^−1^·g^−1^ dry weight when bacterial biofilms were removed by washing. In *Egeria densa*, *Cabomba caroliniana*, and *Chara braunii*, 92–99% of the CH_4_ consumption was lost in the washed samples, whereas submerged parts of aquatic plants with floating or aerial leaves, *Eichhomia cassipes* and *Trapa japonica* retained 61 and 41% of the activity after washing, respectively. These results suggest that most methanotrophs were loosely associated with the surface of aquatic plants, but some associated tightly with or were within aquatic plants.

### Quantitative detection of methanotrophs in aquatic plants

**Figure 3:**
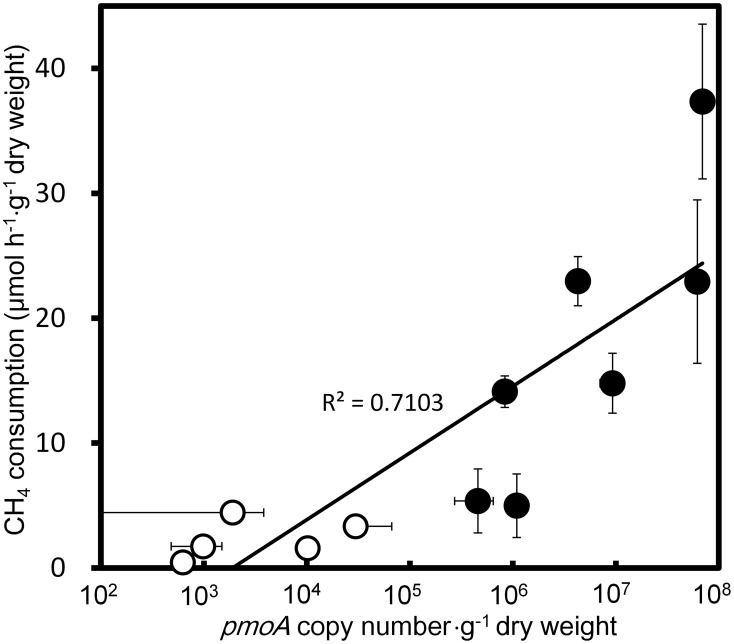
**Correlation of qPCR estimated *pmoA* copy numbers and CH_4_ consumption activities in the aquatic plants.** Filled circles and open circles represent the data from the native and slightly washed samples, respectively. Data represent the mean ± SD from three different samples independently prepared in parallel.

qPCR targeting a component of the pMMO-encoding gene, *pmoA*, produced amplicons of a single size from the extracted DNA of all CH_4_ consuming samples, i.e., *E. densa*, *C. caroliniana*, *C. braunii*, *Potamogeton maackianus*, *Vallisneria natans*, *P. crispus*, and submerged parts of *T. japonica* and *E. cassipes*. The qPCR-estimated copy numbers of *pmoA* from those samples were well correlated (*r*^2^ = 0.71) with the CH_4_ consumption activities (Figure 3), clearly indicating the involvement of methanotrophs in CH_4_ consumption. The populations of methanotrophs in aquatic plants were also variable. In the native sample, the copy numbers of *pmoA* were in the range from 4.6 ± 1.8 × 10^5^ to 6.9 ± 0.77 × 10^7^ copies·g^−1^ dry weight. The *pmoA* copy number declined to 990 ± 510 to 1.0 × 10^4^ ± 890 copies·g^−1^ dry weight after washing, corresponding to 0.001–6.5% of the *pmoA* before washing. As observed with CH_4_ consumption activities, this result also supported a loose association of methanotrophs with the surface of aquatic plants. To date, populations of methanotrophs associated with emergent parts of plants have not been investigated in detail. Our present results indicate that methanotrophs associated with aquatic plants constitute a larger CH_4_ sink than those associated with the phyllosphere of terrestrial plants, and have demonstrated an unexpected contribution of aquatic plants to CH_4_ consumption.

### Phylogeny of methanotrophs associated with aquatic plants

**Figure 4:**
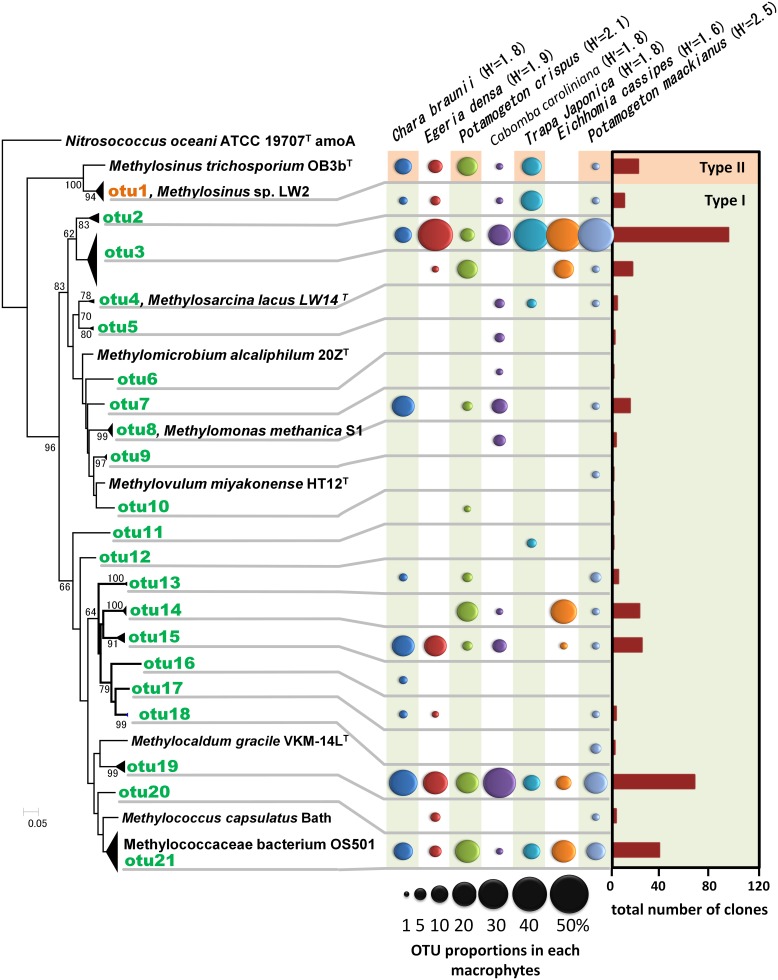
**Neighbor-joining tree of methanotrophic phylotypes detected in the aquatic plants based on the deduced amino acid sequences of *pmoA* genes.** Sequence of *amoA* of *Nitrosococcus oceani* was used as an outgroup to root the tree. H' values are Shannon diversity indices (Shannon and Weaver, 1948). Bootstrap values (>60%) obtained from 1000 resamplings are shown. Accession numbers for the sequences are AAC25091, ACN73467, ACN73466, AAA87220, AAF08211, AAB49821, AAC04380, BAL04120, ABD13901, YP 004915812, BAJ17641, AAA87218, AAG13081.

**Figure 5:**
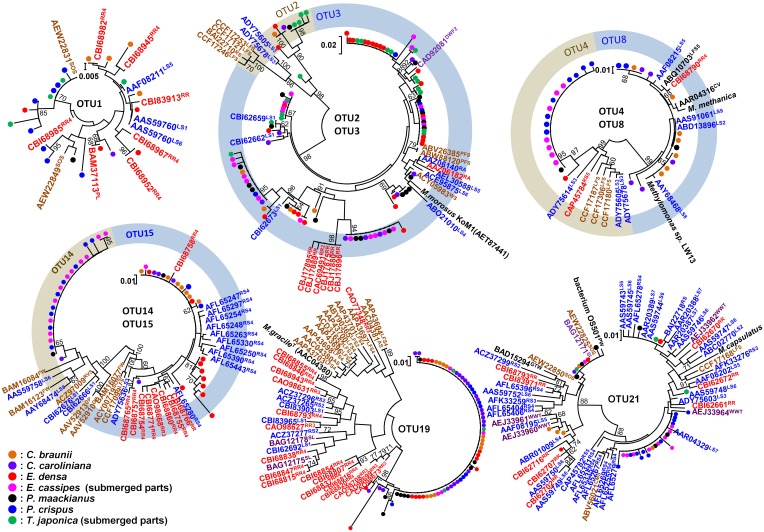
**Neighbor-joining tree of individual clones in abundant OTUs and their related environmental clones based on deduced amino acid sequences of *pmoA* genes.** The clones obtained in this study were indicated as closed circles. Bootstrap values (>60%) obtained from 1000 resamplings are shown. Related clones are indicated as accession numbers with superscript indicating their origins as follows; plants in red including RR, rice root and PL, plant leaf, saturated soil or sediment in blue including RS, rice field soil; LS, lake sediment; RA, river aquifer; TCE, TCE-contaminated aquifer; WS, wetland soil; and RVS, river sediment, unsaturated soil in brown including AS, agricultural soil; PFS, permafrost soil; LFS, landfill-cover soil; SOS, surface soils of onshore oil and gas fields; and CMS, coal mine soil, clone from water in purple including DWF, drinking water facility; SL, methane-consuming sludge; WWT, wastewater treatment plant; DP, drainage of peat land; GTW, subsurface geothermal water stream in a Japanese gold mine, and clones from biomat in black including FWI, freshwater iron-rich microbial mat; PW, pond water; and CV, biomat and water in cave.

A total of 358 cloned *pmoA* gene sequences were determined and classified into 21 OTUs (Figure 4 and Supplemental Table 2). These sequences were similar to those in members of the genus *Methylosinus*, which are type II methanotrophs in the Alphaproteobacteria, and the genera *Methylosarcina*, *Methylomicrobium*, *Methylomonas*, *Methylovulum*, *Methylocaldum*, and *Methylococcus*, which are type I methanotrophs in the Gammaproteobacteria. Three OTUs, OTU3, OTU19, and OTU21 were detected in all aquatic plants and represented 28, 20, and 11% of the total clones, respectively. The sequences of OTU3, the most abundant OTU, were most closely related (94–99% amino acid sequence identity) to *pmoA* sequences of *Methylosarcina lacus* LW14 (Kalyuzhnaya et al., 2005) among the available isolates, and were most closely related to *pmoA*-clones from rice roots (Shrestha et al., 2010), lake littoral wetlands (Siljanen et al., 2011), lake littoral sediments (Deutzmann et al., 2011), and rice field soil (Mayumi et al., 2010) (Figure 5). The sequences of OTU19, the second most abundant OTU, were 87–89% similar to the *pmoA* sequence from *Methylocaldum gracile* VKM-14L (Bodrossy et al., 1997) among isolated strains, and all of the clones were most closely related to *pmoA* clones from rice roots (Shrestha et al., 2008). The sequence of the third most abundant OTU (OTU21), which was 91–95% similar to the *pmoA* sequence of *Methylococcaceae* sp. strain OS501 (Iguchi et al., 2011) as the closest related isolate, was most closely related to a variety of environmental clones from littoral sediments of Lake Constance (Bussmann et al., 2004), CH_4_-consuming sludge (Osaka et al., 2008), and rice roots (Shrestha et al., 2010). In addition to those highly abundant OTUs, six OTUs had more than 5% frequency among the total clones and were detected in more than four aquatic plants as follows; the sequences of OTU1 were closely related to clones from lake sediment (Costello and Lidstrom, 1999; Pester et al., 2004), rice roots (Lüke et al., 2010), plant leaves (Iguchi et al., 2012) and onshore oil and gas field soil (Xu et al., 2013), the sequences of OTU2 were related to clones from landfill soils (Henneberger et al., 2012), the sequences of OTU4, OTU8, and OTU14 were related to clones from lake sediments (Costello and Lidstrom, 1999; Lin et al., 2004; Nercessian et al., 2005; Bussmann et al., 2006; Deutzmann et al., 2011; Siljanen et al., 2011), and the sequences of OTU15 were related to clones from rice roots (Shrestha et al., 2008; Lüke et al., 2010) and rice field soil (Ho et al., 2011).

Among the identified OTUs, all sequences of OTU19 as well as most sequences of OTU15 formed a cluster with the sequences of the clones obtained from rice roots, but not with other clusters. In particular, methanotrophs corresponding to OTU19 were widely and abundantly distributed in seven different aquatic plants and the sequences formed a cluster only with the sequences of the clones obtained from two different rice roots in different places. These results suggest that methanotrophs represented by OTU19 live in association with aquatic plants.

### Enrichment and isolation of methanotrophs from aquatic plants

We attempted to enrich and isolate methanotrophs associated with *C. demersum*, *E. densa*, *E. crassipes*, *T. japonica*, *P. crispus*, and *C. caroliniana*. Since the use of NMS and AMS media could result in biased enrichment of particular methanotrophs (Hoefman et al., 2014), our results are not expected to directly reflect the population of methanotrophs on plant surfaces. Nevertheless, we wanted to isolate a variety of methanotrophs from plant surfaces, and therefore, NMS and AMS media were applied as the first trial. Indeed, our previous study also showed that a novel genus of type-I methanotroph, *Methylovulum miyakonense* (Iguchi et al., 2011) could be isolated by using NMS medium. Each culture that had been inoculated with a piece of an aquatic plant consumed all of the CH_4_ within a week, and maintained CH_4_ consumption activity after several serial transfers. For each sample, 1–9 colonies among 100 colonies grown on agar plates consumed CH_4_, and these colonies were further purified. The 100 picked colonies included 50 colonies from NMS medium and 50 colonies from AMS medium. Finally, 23 strains were isolated; these belonged to the genera *Methylosinus*, and *Methylocystis* based on sequencing of 16S rRNA and *pmoA* genes (Supplemental Figure 1). However, no type-I methanotrophs were isolated despite their abundance in aquatic plants. The nitrogen source used did not seem to result in isolation of specific methanotrophs, as similar strains were obtained using both NMS and AMS. Although there seems to have been a bias in the isolation of the dominant methanotrophs from aquatic plants due to the enrichment method, isolation of methanotrophic strains from aquatic plants would be the first step to reconstructing and establishing an ecological system of aquatic plants and methanotrophs having high CH_4_ consuming activity.

## Discussion

We evaluated CH_4_ consuming activities of methanotrophs associated with different plant species sampled from various terrestrial and aquatic environments. Our study revealed that all aquatic plants from a eutrophic lake showed high CH_4_ consumption activity. The CH_4_ consumption activity (3.7–37 μmol·h^−1^·g^−1^ dry weight) was higher than that of rice roots (0.2–0.4 μmol·h^−1^·g^−1^ dry weight) (Eller and Frenzel, 2001) and roots and rhizomes of emergent aquatic plants (0.1–6.4 μmol·h^−1^·g^−1^ dry weight) (King, 1994) as soil-free epiphytic methanotrophic activities, as well as previous reports of *Sphagnum* moss (Raghoebarsing et al., 2005) and in aquatic plants (Sorrell et al., 2002). To evaluate and control the carbon cycle in aqueous environments, it is important to understand not only the atmospheric carbon cycling but also the local CH_4_ cycling and the derivative food chain in lakes.

Assuming the average CH_4_ consumption of native aquatic plants is common to all aquatic plants though the year, 160 t·y^−1^ of CH_4_ was potentially consumed by 2500 t of aquatic plants in the basin (calculated from 1995 data, Shiga Prefectural Fisheries Experiment Station 1998), which corresponds to 17% of the total CH_4_ emitted (770 t·y^−1^) from the lake surface of the basin (Kagotani et al., 1996). This large potential is suggested to represent substantial local CH_4_ cycling via methanotrophs associated with aquatic plants in this lake. The CH_4_ consumed by microbial communities associated with aquatic plants may have come from both dissolved CH_4_ in the water column and uptake from the sediment. In this lake sediment, 90% of the CH_4_ originating from organic matter in the deep sediment was consumed in the oxic surface (Murase et al., 2005). Therefore, the remaining 10% of the CH_4_ that passed though the oxic surface and was emitted into the water column represents CH_4_ that undergoes local cycling. Another route may be uptake from sediment via plant roots as observed in rice and other rooted aquatic plants. In this study, there were no big differences between unrooted samples, i.e., pieces of *E. densa* and *C. demersum*, and other rooted samples, suggesting that CH_4_ both from the sediment and the water column can be consumed by the aquatic plants. All aquatic plants assayed in this study, which were obtained from a eutrophic basin, consumed CH_4_, while aquatic plants sampled from oligotrophic environments did not (Sorrell et al., 2002). The *Km* for CH_4_ consumption in aquatic plants was reported to be in the range 3–6 μmol· L^−1^ (Sorrell et al., 2002), and this is not sufficient for uptake of atmospheric CH_4_. From this, we speculate that most of the CH_4_ utilized by methanotrophs in aquatic plants was produced in the sediment and taken up via the water column and roots.

In addition, methanotrophs corresponding to OTU19, which is related to *Methylocaldum*, may be selected through interaction with the plant surface. OTU19 was detected in all aquatic plants and the sequences clustered with clones from rice roots from two different sources (Shrestha et al., 2008; Lüke et al., 2010). The detection of OTU19 from different species of plants and the distinct clustering from other environmental clones suggests a particularly strong association of these methanotrophs with plant environments.

In conclusion, this study has shed light on the high potential of methanotrophs associated with aquatic plants as a local CH_4_ sink. We identified methanotrophs responsible for CH_4_ consumption as diverse uncultured type I methanotrophs in the gammaproteobacterial lineage, which were related to methanotrophs detected from rice roots, lake sediments, and rice fields.

## Author contributions

Naoko Yoshida performed all experiments and drafted the manuscript. Hiroyuki Iguchi and Akio Murakami participated in data interpretation and revising the draft. Hiroya Yurimoto and Yasuyoshi Sakai supervised the work.

## Conflict of interest statement

The authors declare that the research was conducted in the absence of any commercial or financial relationships that could be construed as a potential conflict of interest.
