# Supplementary material for: Aquatic plant surface as a niche for methanotrophs
Source: Front Microbiol. 2014 Feb 3;5:30. doi: 10.3389/fmicb.2014.00030 (PMC3909826; doi:10.3389/fmicb.2014.00030)
Supplement: Supplementary file 1 [file DataSheet1.DOCX]

**Supplemental Table 1. The list of plant and macrophytic algal species assayed in the first screen for high CH_4_ consuming plants**

| no | Life form | Species | Inoculated tissues | Sampling place |
| --- | --- | --- | --- | --- |
| 1 | Submerged aquatic plant | *Egeria densa* | leaf and stem | Lake Biwa^*2^ |
| 2 |  | *Cabomba caroliniana* | leaf and stem | Lake Biwa |
| 3 |  | *Chara braunii* (characean alga) | thallus | Lake Biwa |
| 4 |  | *Potamogeton maackianus* | leaf and stem | Lake Biwa |
| 5 |  | *Elodea nuttallii* | leaf and stem | Lake Biwa |
| 6 |  | *Vallisneria natans* | leaf and stem | Lake Biwa |
| 7 |  | *Potamogeton crispus* | leaf and stem | Lake Biwa |
| 8 |  | *Ceratophyllum demersum* | leaf and stem | Lake Biwa |
| 9 | Aquatic plant with floating or aerial leaves | *Trapa Japonica* | leaf, stem and root | Lake Biwa |
| 10 |  | *Eichhomia cassipes* | leaf, stem and root | Lake Biwa |
| 11 | Emergent plant in shallow water | *Aeschynomene indica* | S/E^*1^ | Lake Biwa |
| 12 |  | *Nelumbo nucifera* | S/E | Lake Biwa |
| 13 |  | *Myriophyllum aquaticum* | S/E | Lake Biwa |
| 14 |  | *Acorus calamus* | S/E | Lake Biwa |
| 15 |  | *Typha latifolia* | S/E | Lake Biwa |
| 16 |  | *Phragmites australis* | S/E | Lake Biwa |
| 17 |  | *Ludwigia epilobioides* | S/E | paddy field^*3^ |
| 18 |  | *Monochoria vaginalis* | S/E | paddy field |
| 19 |  | *Schoenoplectus triqueter* | S/E | paddy field |
| 20 |  | *Oryza sativa* | S/E | paddy field |
| 21 | Woody plant | *Camellia japonica* | leaf | botanical garden^*4^ |
| 22 |  | *Photinia glabra* | leaf | botanic garden |
| 23 |  | *Carpinus tschonoskii* | leaf | botanic garden |
| 24 |  | *Quercus myrsinifolia* | leaf | botanic garden |
| 25 |  | *Euonymus hamiltonianus* | leaf | botanic garden |
| 26 |  | *Aucuba japonica* | leaf | botanic garden |
| 27 |  | *Machilus thunbergii* | leaf | botanic garden |
| 28 |  | *Cinnamomum tenuifolium* | leaf | botanic garden |
| 29 |  | *Celtis sinensis* | leaf | botanic garden |
| 30 |  | *Neolitsea sericea* | leaf | botanic garden |
| 31 | Macrophytic marine algae and sea grass | *Sargassum fluvellum* (brown alga) | thallus | Seto Inland Sea^*5^ |
| 32 |  | *Sargassum yamamotoi* (brown alga) | thallus | Seto Inland Sea |
| 33 |  | *Ulva pertusa* (green alga) | thallus | Seto Inland Sea |
| 34 |  | *Halarachnion latissimum* (red alga) | thallus | Seto Inland Sea |
| 35 |  | *Schizymenia dubyi* (red alga) | thallus | Seto Inland Sea |
| 36 |  | *Grateloupia lanceolata* (red alga) | thallus | Seto Inland Sea |
| 37 |  | *Padina arborescens* (brown alga) | thallus | Seto Inland Sea |
| 38 |  | *Zostera marina* (sea grass) | leaf | Seto Inland Sea |
| 39 |  | *Colpomenia sinuosa* (brown alga) | thallus | Seto Inland Sea |

*^1^: S/E indicates the submerged part, *i.e.* mixture of submerged stem and root, or emergent part, *i.e.* mixture of emergent stem and leaf.

*^2^: Shallow eutrophic sub-basin of Lake Biwa in Japan (35°4’ 32”N, 135°56’5”E).

*^3^: A paddy field in Kusatsu, Shiga, Japan (35°2’51” N, 135°56’11” E).

*4: Botanical garden in Kyoto University‎, Kyoto, Japan (35°1’49” N, 135°47’12” E).

*5: Iwaya port (34°35’ 22”N, 135°0’24”E) and Oiso coast (34°33’ N,135°0’E) in Awaji Island, Japan.
