# Supplementary material for: Aquatic plant surface as a niche for methanotrophs
Source: Front Microbiol. 2014 Feb 3;5:30. doi: 10.3389/fmicb.2014.00030 (PMC3909826; doi:10.3389/fmicb.2014.00030)
Supplement: Supplementary file 2 [file DataSheet2.DOCX]

**Supplemental Table 2. List of clones classified into each OTU and PmoA amino acid sequence similarity with the most closely related strains.** The clones designated HG and BQ were obtained from *C. caroliniana*. Other clones sources were as follows; OK: *E. densa*, IB: *C. braunii*, SS: *P. maackianus*, EB: *P. crispus*, HS: submerged parts of *T. Japonica*, HT: submerged parts of *E. cassipes*.

| OTU | Clones | Most closely related strain | Amino acid similarity (%) |
| --- | --- | --- | --- |
| 1 | HG2-34,OK-05,OK-22,IB-14,HS-22,IB-35,IB-29,OK-19,IB-41,OK-27,EB-11,EB-15,HS-26,EB-52,EB-75,HS-25,EB-99,HS-05,SS-23,EB-44,EB-70,EB-06 | *Methylosinus* sp. LW2 (AAF08211) | 94-99 |
| 2 | BQ661-6,IB-08,OK-03,SS-64,HS-56,HS-46,HS-54,HS-48,OK-18,HS-50 | *Methylosarcina lacus* LW14^T^ (AAG13081) | 84-86 |
| 3 | HG-72,HT-72,SS-81,SS-74,HG-61,HT-67,HT-81,HG-67,HT-74,SS-88,OK-15,HT-101,HG-57,HG-44,HT2-26,EB-82,SS-10,HT2-26,SS-05,HT2-47,HT2-46,IB-12,SS-09,HT2-34,HT2-48,HS-51,SS-54,IB-23,OK2-11,OK2-08,OK-59,HT-60,HT-60,OK-21,IB45,SS-66,HG-60,SS-56,BQ661-10,SS-47,HS-15,OK2-16,SS-80,EB-13,HT2-45,EB-29,SS-13,OK-58,HT-66,SS-06,HT2-03,SS-16,HS-24,OK-64,OK2-17,OK2-10,SS-57,SS-72,SS-44,SS-24,HT2-49,HT2-11,OK2-12,HT2-51,HT-64,HS-29,HG2-46,SS-02,OK-60,OK-57,OK-52,OK-55,OK-53,HS-23,HS-17,OK-51,OK2-14,OK2-15,OK2-13,HS-07,HS-16,EB-34,OK-29,HS-31,HS-19,HS-47,HS-36,HT2-21,HS-27,OK-63,HT2-16,HG-58,IB-20,BQ661-b41,OK2-06,SS-79,SS-59,SS-55,OK2-19 | *Methylosarcina lacus* LW14^T^ (AAG13081) | 86-87 |
| 4 | SS-15,HT2-02,HT2-37,HT2-32,HT2-22,EB-04,EB-101,EB-86,HT2-19,EB-14,HT2-08,HT2-01,EB-31,EB-100,EB-10,EB-95,OK-14 | *Methylosarcina lacus* LW14^T^ (AAG13081) | 88 |
| 5 | HG2-35,BQ661-5,SS-67,HS-52 | *Methylosarcina lacus* LW14^T^ (AAG13081) | 91 |
| 6 | BQ661-b4,BQ661-4 | *Methylobacter tundripaludum* SV96^T^ (JH109152) | 89 |
| 7 | HG2-50 | *Methylobacter tundripaludum* SV96^T^ (JH109152) | 92 |
| 8 | HG-73,HG2-49,IB-37,EB-07,EB-74,HG-63,IB-31,IB-24,IB-01,SS-18,IB-05,IB-09,IB-04,HG-66,BQ661-b47 | *Methylomonas methanica* S1 (AAA87218) | 94-96 |
| 9 | BQ661-b44,HG2-33,BQ661-b11 | *Methylomonas methanica* S1 (AAA87218) | 88-89 |
| 10 | SS-70 | *Methylobacter tundripaludum* SV96^T^ (EGW23568) | 92 |
| 11 | EB-33 | *Methylococcus capsulatus* Bath (AAB49821) | 87 |
| 12 | HS-43 | *Methylococcus capsulatus* Bath (AAB49821) | 89 |
| 13 | SS-86,SS-22,EB-78,IB43,EB-89 | *Methylococcus capsulatus* Bath (AAB49821) | 85 |
| 14 | BQ661-1,SS-04,EB-102,HT-84,HT-85,EB-96,HT-99,EB-03,EB-17,HT-86,EB-93,HT-84,HT-69,EB-94,HT-82,HT2-29,EB-27,HT-83,HT-77,EB-02,HT-96,HT-95,EB-77 | *Methylococcus capsulatus* Bath (AAB49821) | 81-83 |
| 15 | HG-70,BQ661-b42,HG2-47,IB-28,BQ661-11,IB-40,OK-13,IB-33,SS-03,IB-18,IB-11,OK-28,IB-25,IB-15,EB-80,EB-25,HT2-06,OK-12,OK-01,OK-02,OK-11,OK-10,OK-06,OK-04,OK-08 | *Methylococcus capsulatus* Bath (AAB49821) | 86-88 |
| 16 | IB-13 | *Methylococcus capsulatus* Bath (AAB49821) | 80 |
| 17 | IB-06,SS-01,OK-09 | *Methylococcus capsulatus* Bath (AAB49821) | 85 |
| 18 | SS-17,SS-71 | *Methylococcus capsulatus* Bath (AAB49821) | 85 |
| 19 | HG-71,HG-53,HT2-53,IB-42,BQ661-b43,SS-82,HG-52,SS-50,OK-30,IB-32,EB-32,HG-59,HG2-28,OK-20,EB-83,SS-65,OK-26,SS-48,IB46,OK-56,IB-34,IB-19,EB-22,IB-17,HT-63,HS-20,HG-65,OK2-07,HS-42,HG-64,EB-24,HG2-30,HG-54,HG-45,EB-19,OK-62,HG2-27,OK-16,HG2-24,EB-91,SS-63,BQ661-8,SS-53,EB-01,HS-08,BQ661-b31,HT2-33,EB-83,HG2-31,IB-39,SS-83,EB-08,OK2-09,IB48,HT2-31,OK-25,IB44,HG2-10,IB-38,IB-10,BQ661-12,BQ661-7,OK2-02,HG-62,HG2-26,SS-62,OK2-18,SS-89,EB-53,HG2-11,OK-24 | *Methylocaldum gracile* VKM-14LT (AAC04380) | 87-89 |
| 20 | SS-45,OK-17,OK-61 | *Methylocaldum gracile* VKM-14L^T^ (AAC04380) | 78-79 |
| 21 | HG2-48,HS-39,IB-36,SS-08,HT2-28,OK2-05,SS-58,SS-69,OK2-04,IB-27,IB-16,IB-26,SS-51,HT2-24,EB-21,SS-11,IB-30,EB-37,HT2-23,EB-87,EB-40,HT2-54,HT2-41,EB-76,EB-38,HT2-18,EB-97,HT2-14,HT2-39,HT2-12,EB-09,EB-16,EB-39,EB-23,HT2-30,HS-38,EB-98,SS-12,OK-23,HS-21 | *Methylococcaceae bacterium* OS501 (BAL04120) | 91-99 |
