# Supplementary figures and images for: Aquatic plant surface as a niche for methanotrophs
Source: Front Microbiol. 2014 Feb 3;5:30. doi: 10.3389/fmicb.2014.00030 (PMC3909826; doi:10.3389/fmicb.2014.00030)

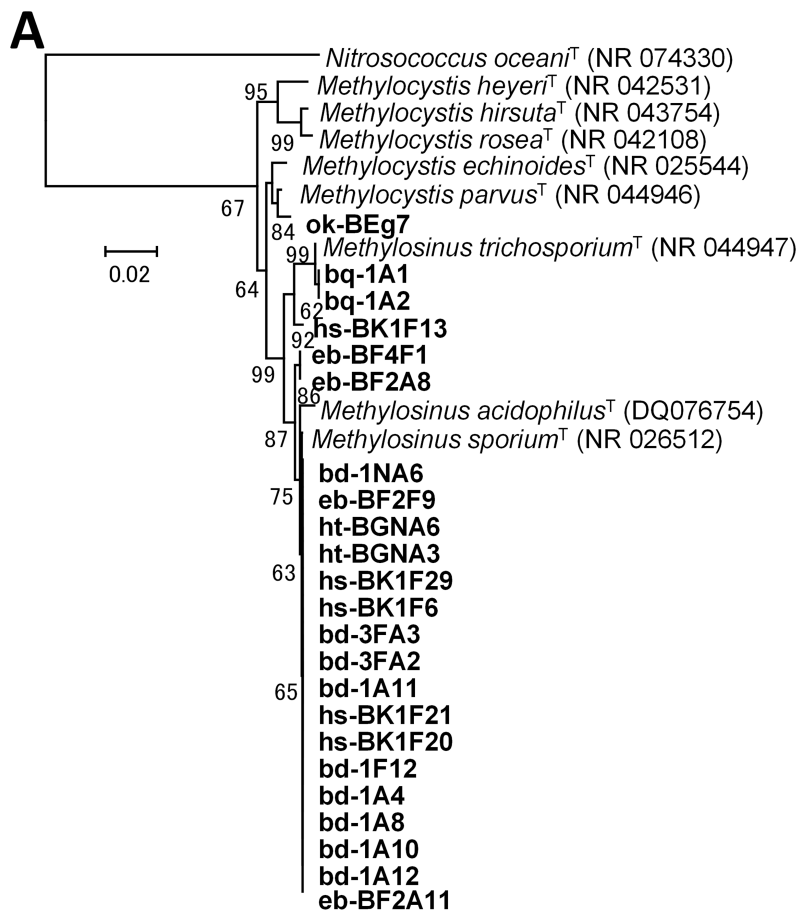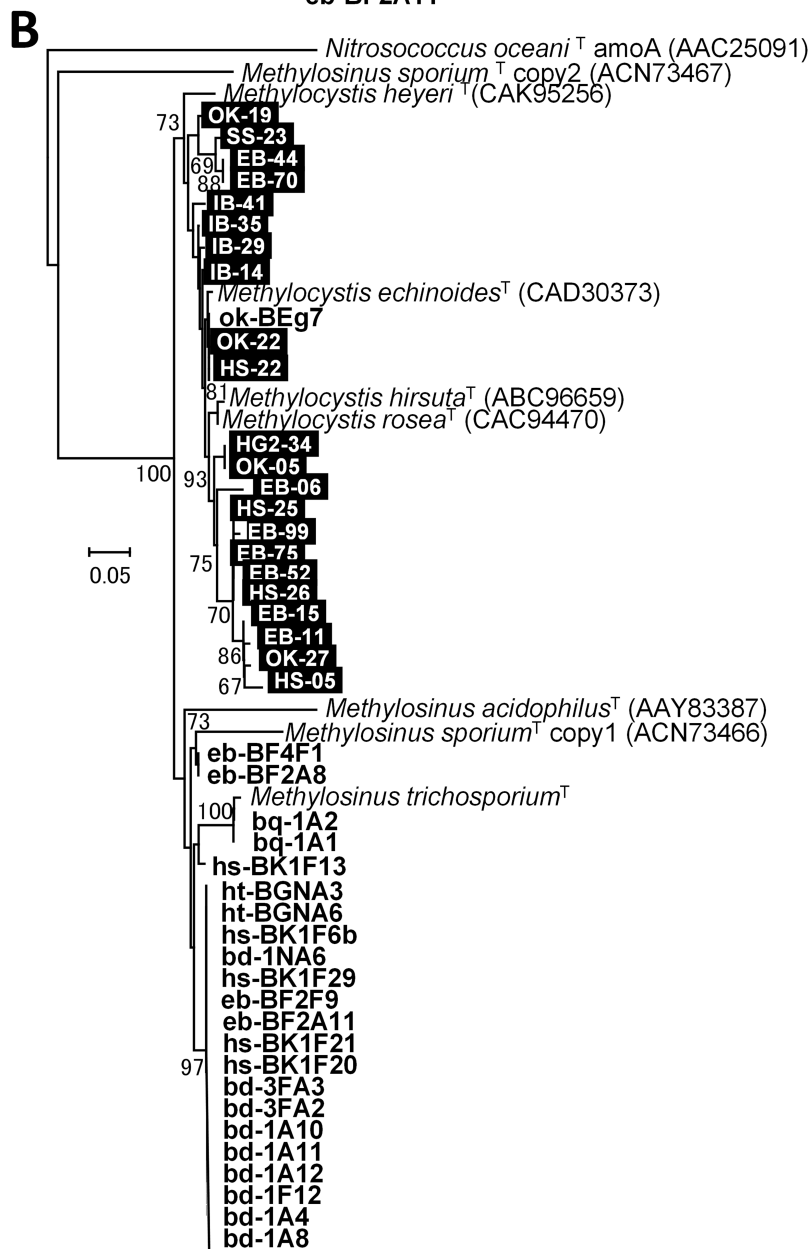

Supplement: Supplementary file 3 [file Presentation1.PDF]
