# Supplementary material for: Aquatic plant surface as a niche for methanotrophs
Source: Front Microbiol. 2014 Feb 3;5:30. doi: 10.3389/fmicb.2014.00030 (PMC3909826; doi:10.3389/fmicb.2014.00030)
Supplement: Supplementary file 4 [file Presentation2.PDF]

Supplemental Figure 1. Neighbor-joining tree of methanotrophic strains isolated from the aquatic plants based on 16S rRNA gene sequences (A) and amino acid sequences of PmoA (B). The sequences in bold correspond to the strains isolated from each plant, and their origins are as follows; bd-: *C. demersum*, ok-: *E. densa*, ht-: *E. crassipes*, hs-: *T. japonica*, bq-: *C. caroliniana*, eb-: *P. crispus*. The sequences in white were clones of OTU1. Bootstrap values (>60%) obtained from 1,000 resamplings are shown.
